# Systemic factors associated with poor anatomical response to anti-VEGF therapy in patients with diabetic macular edema: evidence from a large clinical cohort

**DOI:** 10.3389/fendo.2026.1848557

**Published:** 2026-07-21

**Authors:** Xinru Jia, Yan Shi, Mengxiang He, Dongwei Yao, Chengfei Lin

**Affiliations:** 1 Department of Ophthalmology, The First Affiliated Hospital of Ningbo University, Ningbo, Zhejiang, China; 2 Ningbo Haishu Taixue Eye Clinic, Ningbo, Zhejiang, China

**Keywords:** anti-VEGF, central macular thickness, conbercept, diabetic macular edema, generalized estimating equation, HbA1c

## Abstract

**Background:**

Diabetic macular edema (DME) is a leading cause of vision impairment in the working-age population worldwide. Although intravitreal anti-vascular endothelial growth factor (anti-VEGF) therapy represents the established first-line treatment, a substantial proportion of patients exhibit inadequate anatomical responses. This retrospective cohort study aimed to identify independent systemic factors associated with poor anatomical response to intravitreal Conbercept in patients with DME.

**Methods:**

In this retrospective cohort study, 1,039 eyes from 691 patients with DME were analyzed. All patients underwent a standardized loading phase of intravitreal Conbercept (0.5 mg) administered monthly for three consecutive months. The primary outcome was the absolute and percentage change in central macular thickness (CMT) from baseline to one month after the third injection. Eyes achieving a percentage reduction in CMT exceeding 10% from baseline were classified as “responders.” Generalized Estimating Equation (GEE) models were employed to identify independent factors while accounting for the intra-subject correlation between bilateral eyes.

**Results:**

Following the three-month Conbercept loading phase, the median CMT decreased significantly from 325.00 μm (IQR: 281.00–448.00 μm) at baseline to 291.00 μm (IQR: 268.00–334.00 μm) at the three-month follow-up, representing a median absolute reduction of 17.00 μm (P < 0.001). Concurrently, median best-corrected visual acuity (BCVA) improved from 0.30 (IQR: 0.15–0.50) to 0.40 (IQR: 0.25–0.50) (P < 0.001). Based on the responder criterion, 431 eyes (41.48%) were classified as responders and 608 eyes (58.52%) as non-responders. In the multivariable linear GEE model, baseline CMT, baseline BCVA, LDL-C, fasting blood glucose, and HbA1c were independently associated with degree of CMT reduction. In the multivariable logistic GEE model, lower baseline CMT (Estimate = −0.008, P < 0.001), higher baseline BCVA (Estimate = 5.274, P < 0.001), elevated fasting blood glucose (Estimate = 0.048, P = 0.030), and elevated HbA1c (Estimate = 0.192, P < 0.001) were independently associated with poor anatomical response.

**Conclusion:**

Systemic metabolic status—particularly HbA1c and fasting glucose levels—and baseline ocular morphology are independently associated with of anatomical response to intravitreal Conbercept in DME. These findings highlight the clinical imperative for integrating systemic metabolic optimization into the management strategy for DME patients prior to and during anti-VEGF loading therapy.

## 1 Introduction

Diabetic retinopathy (DR), of which diabetic macular edema (DME) is the principal vision-threatening manifestation, affects approximately 30% to 40% of all individuals with diabetes (1) and represents a leading cause of vision impairment in the working-age population globally (2). With the escalating global prevalence of diabetes mellitus, projected to affect 783 million people by 2045, the clinical burden of DME is expanding at an unprecedented rate (3). In China, DR represents a critical public health burden, with significant regional disparities in prevalence, screening coverage, and access to management (2). The development of DME is a complex process primarily driven by chronic hyperglycemia, which induces microvascular damage and promotes disease progression through multiple interacting metabolic pathways (4). Prolonged diabetes duration and poorly controlled glycated hemoglobin (HbA1c) are well-established systemic risk factors that accelerate the transition from non-proliferative retinopathy to vision-threatening macular edema (5).

The core pathophysiology of DME involves the breakdown of the blood-retinal barrier (BRB) (6). Chronic high glucose levels trigger the overexpression of vascular endothelial growth factor (VEGF), which leads to increased vascular permeability and the subsequent accumulation of intraretinal and subretinal fluid (6). Consequently, intravitreal injection of anti-VEGF agents has been established as the first-line therapeutic strategy (7, 8). Landmark RCTs, including Protocol T, which established the comparative efficacy among anti-VEGF agents (9), and the VIVID/VISTA studies, which demonstrated the superiority of anti-VEGF therapy over laser photocoagulation in both functional and anatomical outcomes (10), have fundamentally shifted the treatment paradigm for DME. Specifically, the VIVID and VISTA trials demonstrated that intravitreal aflibercept led to significant and durable gains in BCVA—with mean BCVA gains ranging from 10.3 to 11.7 letters across both studies and both dosing regimens—and robust reductions in central subfield thickness that were sustained through 148 weeks, regardless of baseline edema severity (10). Complementing these global findings, the SAILING study in China has provided localized evidence for the efficacy of Conbercept, a domestically developed decoy receptor (11). Unlike traditional monoclonal antibodies, Conbercept is a recombinant fusion protein that incorporates the second immunoglobulin (Ig) domain of VEGF receptor 1 (VEGFR-1) and the third and fourth Ig domains of VEGFR-2. This unique molecular configuration enables it to bind with higher affinity to all isoforms of VEGF-A, VEGF-B, and placental growth factor (PlGF). The SAILING study confirmed that Conbercept administered as needed (PRN) resulted in a mean BCVA improvement of 8.2 letters at 12 months, significantly outperforming laser therapy and reinforcing its role as a pivotal first-line intervention in the Chinese clinical setting (11).

However, a critical gap remains in the real-world management of DME. Despite regular treatment, a substantial proportion of patients exhibit an inadequate anatomical response to anti-VEGF agents; real-world survival analyses indicate that undesirable treatment outcomes develop in approximately 50% of DME patients within 2.3 years of initiating therapy (12). These “non-responders” often experience persistent macular thickening, and structural disorganization of the retinal inner layers has been shown to predict progressive and potentially irreversible visual acuity decline (13). While ocular biomarkers—such as the integrity of the inner segment–outer segment junction and the presence of epiretinal membranes—have been extensively investigated as predictors of anti-VEGF response (14), existing evidence regarding the impact of systemic risk factors on anatomical outcomes remains inconsistent and inconclusive. Many studies are further limited by small sample sizes, a lack of standardized response definitions, or the failure to account for the correlation between bilateral eyes in statistical modeling, all of which hinder the translation of findings into actionable clinical guidelines (15).

This study was conducted in accordance with the Strengthening the Reporting of Observational Studies in Epidemiology (STROBE) guidelines (16). Utilizing a large clinical cohort of 691 patients (1,039 eyes), we aimed to identify independent systemic risk factors—including metabolic indicators such as HbA1c and lipid profiles—that predict a poor anatomical response to Conbercept. By employing Generalized Estimating Equation (GEE) models, this research provides robust evidence to support personalized treatment strategies and prognostic assessments for patients with DME.

## 2 Methods

### 2.1 Study design and participant selection

**Figure 1:**
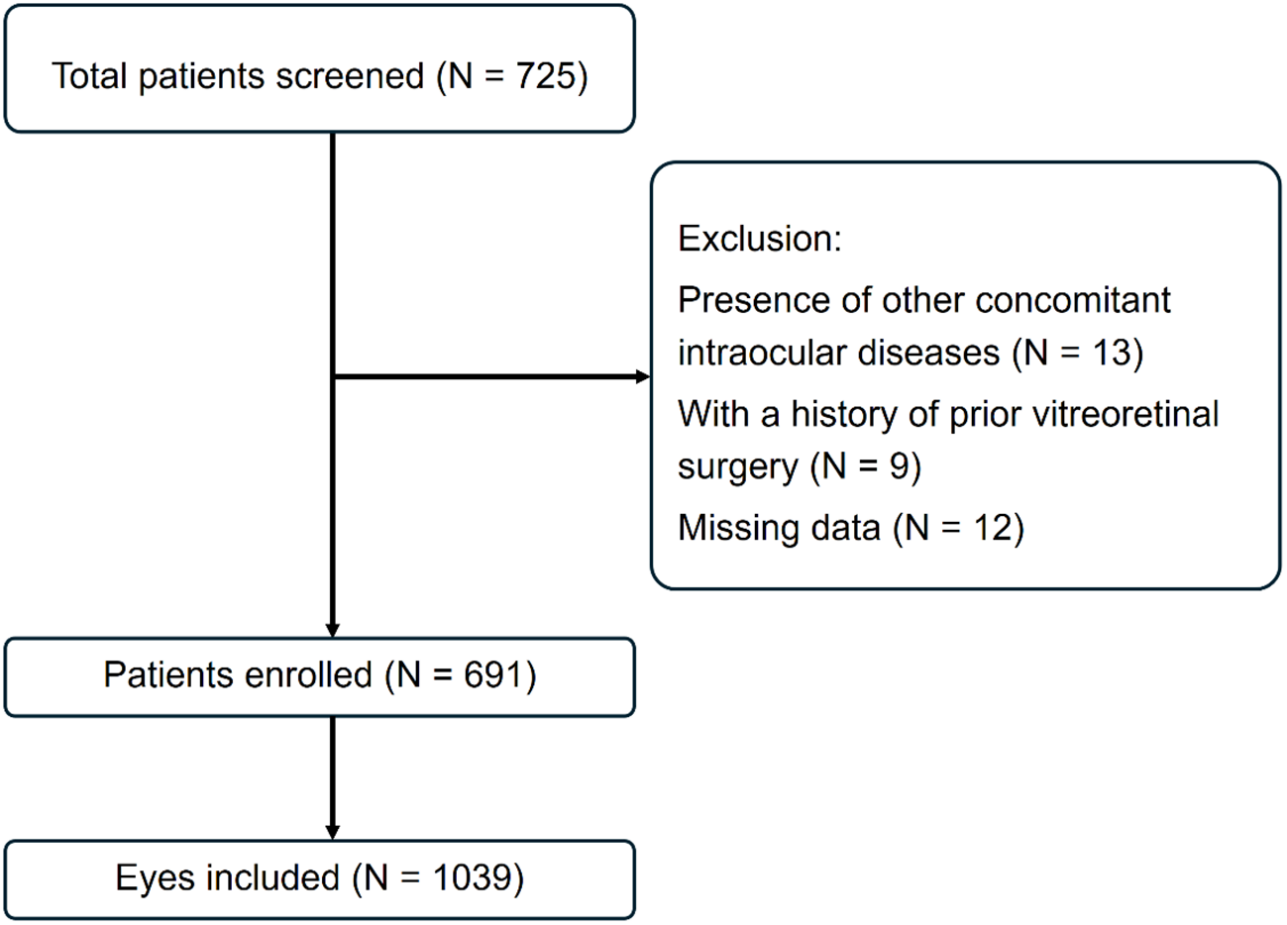
Flowchart of participant selection and enrollment for the study cohort.

This study was designed as a retrospective, large-scale clinical cohort study aimed at identifying predictors of anatomical response in patients with DME. The study protocol adhered to the tenets of the Declaration of Helsinki. A total of 724 patients were initially screened from the clinical database. The selection process, including reasons for exclusion, is detailed in the participant flow chart (**Figure 1**).

#### 2.1.1 Inclusion CRITERIA

Patients were eligible for inclusion if they met the following criteria: 1) Diagnosis of type 2 diabetes mellitus (T2DM) according to the World Health Organization (WHO) diagnostic criteria. 2) Confirmed diagnosis of DME through slit-lamp biomicroscopy and optical coherence tomography (OCT). 3) Baseline central macular thickness (CMT) > 250 μm as measured by Spectralis OCT (Heidelberg Engineering GmbH, Heidelberg, Germany).

#### 2.1.2 Exclusion criteria

Potential participants were excluded based on the following criteria: 1) Prior history of vitreoretinal surgery or the presence of epiretinal membranes (ERM) requiring pars plana vitrectomy (PPV). 2) Concomitant ocular diseases, including glaucoma, intraocular tumors, or congenital eye diseases. 3) High myopia, defined as a refractive error ≥6D. 4) Significant media opacities (e.g., advanced cataract) that precluded high-quality fundus examination or OCT imaging. 5) Incomplete clinical records or absence of required follow-up data after treatment.

### 2.2 Treatment regimen and injection procedure

All eligible eyes underwent a standardized initial loading phase of anti-VEGF therapy, consisting of three consecutive monthly intravitreal injections. The pharmacological agent administered was Conbercept ophthalmic injection (10 mg/mL, 0.05 mL, Chengdu Kanghong Biotech Co., Ltd., China).

Intravitreal Injection Procedure: All injections were performed by experienced ophthalmologists under strictly sterile conditions in an operating room. Following topical anesthesia and thorough disinfection of the periocular skin and conjunctival sac with povidone-iodine, a 30G needle was used to deliver 0.5 mg (0.05 mL) of Conbercept into the vitreous cavity through the pars plana, approximately 3.5 - 4.0 mm posterior to the corneal limbus. Postoperatively, patients were routinely prescribed topical antibiotic eye drops for one week to prevent the occurrence of endophthalmitis.

### 2.3 Clinical evaluation and data collection

#### 2.3.1 Anatomical assessment and primary outcome

The primary outcome of this study was the absolute and percentage change in Central Macular Thickness (CMT) from baseline to one month after the third intravitreal injection (the three-month follow-up). Anatomical response was evaluated using Optical Coherence Tomography (OCT). CMT was defined as the average thickness of the neurosensory retina within the central 1 mm diameter circle centered on the fovea. Because this endpoint reflects the initial loading-phase response, it should not be interpreted as evidence of long-term treatment durability.

#### 2.3.2 Systemic data and comorbidities

Comprehensive systemic data were extracted from the electronic medical record (EMR) system to analyze their correlation with anatomical treatment efficacy. The following variables were collected. Demographic Characteristics: Patient age and gender. Systemic Comorbidities: Presence of hypertension and coronary artery disease (CAD).

#### 2.3.3 Laboratory measurements and metabolic indicators

All biochemical markers were obtained from fasting peripheral blood samples collected within one week prior to the initial anti-VEGF injection. The metabolic profile included: 1) Glycemic Control: Fasting blood glucose and glycated hemoglobin A1c (HbA1c). 2) Lipid Profiles: Total cholesterol (TC), triglycerides (TG), high-density lipoprotein cholesterol (HDL-C), and low-density lipoprotein cholesterol (LDL-C).

### 2.4 Statistical analysis

Statistical analyses were performed to identify systemic predictors of anatomical response to anti-VEGF therapy while strictly accounting for the hierarchical nature of the data. For continuous variables, the Kolmogorov-Smirnov (KS) test was initially applied to assess the normality of the distribution. Normally distributed data were expressed as mean ± standard deviation (SD), whereas non-normally distributed data were presented as medians with interquartile ranges (IQR: 25th–75th percentiles). Categorical variables were summarized as frequencies and percentages. To compare baseline characteristics between groups, the Student’s t-test or Mann-Whitney U test was utilized for continuous data, and the Chi-square test was employed for categorical data.

A critical statistical consideration in ophthalmological research is the inherent correlation between the left and right eyes of the same patient. To address this intra-subject dependency and prevent the inflation of Type I errors, we utilized Generalized Estimating Equation (GEE) models for all regression analyses.

The analysis was conducted in two primary stages. Analysis of CMT Reduction: The total dataset of 1,038 eyes was analyzed using linear GEE models to determine whether the change in central macular thickness (CMT) varied according to clinical characteristics. Initially, univariable GEE models were performed to screen for potential risk factors. Variables demonstrating statistical significance (P < 0.05) in the univariable analysis were subsequently entered into a multivariable GEE model to identify independent predictors of anatomical improvement.

Analysis of Treatment Response (Responder Status): Patients were dichotomized based on their anatomical outcome at the three-month follow-up. A study eye was defined as a “responder” if the percentage reduction in CMT was greater than 10% from baseline; otherwise, it was classified as a “non-responder”. To identify the factors associated with poor response, a logistic GEE model was constructed with treatment response (responder vs. non-responder) as the dependent variable.

Multicollinearity among systemic factors (specifically between fasting glucose and HbA1c) was assessed, and the multivariable models were adjusted accordingly to ensure the stability of the estimates. All statistical tests were two-tailed, and a P-value < 0.05 was considered statistically significant.

## 3 Results

### 3.1 Patient recruitment and enrollment

A total of 725 patients were initially identified from the institutional clinical database and screened for eligibility. Following the systematic application of pre-specified inclusion and exclusion criteria, 34 patients were excluded prior to final enrollment. The reasons for exclusion were as follows: 13 patients were excluded due to the presence of concomitant intraocular diseases; 9 patients were excluded owing to a prior history of vitreoretinal surgery; and the remaining 12 patients were excluded because of incomplete post-treatment follow-up data. The complete participant selection process is illustrated in **Figure 1**. Ultimately, a final cohort of 691 patients was enrolled, contributing a total of 1,039 eyes for analysis. All 1,039 eyes completed the three-month loading-phase treatment protocol and were included in the primary statistical analyses.

### 3.2 Baseline patient characteristics and treatment outcomes

#### 3.2.1 Baseline demographic and clinical characteristics

The baseline demographic and clinical characteristics of the 691 enrolled patients are summarized in **Table 1**. The mean age of the cohort was 59.50 ± 12.50 years, with a slight male predominance (53.26%, n = 368) over female patients (46.74%, n = 323). All 691 patients carried a confirmed diagnosis of type 2 diabetes mellitus. Nearly half of the cohort had comorbid hypertension (48.19%, n = 333), and coronary artery disease (CAD) was present in 19.97% (n = 138) of patients.

**Table 1:** Baseline demographic, systemic, and metabolic characteristics of the study cohort (N = 691 patients).

Variable	N = 691
Age (years), mean ± sd	59.50 ± 12.50
Gender, n (p%)	
Female	323.00 (46.74%)
Male	368.00 (53.26%)
LDL-C (mM), mean ± sd	3.19 ± 1.04
HbA1c (%), mean ± sd	8.87 ± 3.01
TC (mM), median (p25 - p75)	4.93 (4.11 - 5.90)
TG (mM), median (p25 - p75)	1.48 (1.03 - 2.25)
Glucose (mM), median (p25 - p75)	9.58 (6.80 - 13.96)
HDL-C (mM), median (p25 - p75)	1.18 (1.01 - 1.40)
Diabetes, n (p%)	691.00 (100.00%)
CAD, n (p%)	138.00 (19.97%)
Hypertension, n (p%)	333.00 (48.19%)

Data are presented as mean ± standard deviation (sd) for normally distributed continuous variables, and as median with interquartile range (IQR: 25th–75th percentiles) for non-normally distributed continuous variables. Categorical variables are expressed as frequency (n) and percentage (%). CAD, Coronary Artery Disease; TC, Total Cholesterol; TG, Triglycerides; HDL-C, High-Density Lipoprotein Cholesterol; LDL-C, Low-Density Lipoprotein Cholesterol; HbA1c, Glycated Hemoglobin A1c. All patients were diagnosed with type 2 diabetes mellitus.

With respect to metabolic indicators, glycemic control was suboptimal across the cohort. According to the clinical consensus for type 2 diabetes management, HbA1c < 7.0% is defined as well-controlled glycemia, while HbA1c ≥ 7.0% indicates poor glycemic control. The cohort had a mean HbA1c of 8.87 ± 3.01% and a median fasting blood glucose of 9.58 mM (IQR: 6.80–13.96 mM), both substantially exceeding recommended therapeutic targets. Regarding lipid profiles, the mean LDL-C was 3.19 ± 1.04 mM, while median TC and TG were 4.93 mM (IQR: 4.11–5.90 mM) and 1.48 mM (IQR: 1.03–2.25 mM), respectively. Median HDL-C was 1.18 mM (IQR: 1.01–1.40 mM).

#### 3.2.2 Anatomical and functional outcomes following anti-VEGF loading phase

The changes in CMT and BCVA across all 1,039 eyes before and after the three-month loading-phase treatment are presented in **Table 2**. At baseline, the median CMT was 325.00 μm (IQR: 281.00–448.00 μm), reflecting substantial macular thickening consistent with clinically significant DME. Following three consecutive monthly intravitreal injections of Conbercept, the median CMT decreased significantly to 291.00 μm (IQR: 268.00–334.00 μm), corresponding to a median absolute reduction of 17.00 μm (IQR: −100.00 to 4.00 μm; P < 0.001).

**Table 2:** Changes in central macular thickness (CMT) and best-corrected visual acuity (BCVA) before and after anti-VEGF loading-phase treatment (N = 1,039 eyes).

CMT variable (N = 1039)		BCVA variable (N = 1039)	
CMT baseline (μm), median (p25 - p75)	325.00 (281.00 - 448.00)	BCVA baseline, median (p25 - p75)	0.30 (0.15 – 0.50)
CMT after treatment for three months (μm), median (p25 - p75)	291.00 (268.00 - 334.00)	BCVA after treatment for three months, median (p25 - p75)	0.40 (0.25 – 0.50)
CMT difference (μm), median (p25 - p75)	-17.00 (-100.00 - 4.00)	BCVA difference, median (p25 - p75)	0.00 (0.00, 0.20)
p-value	< 0.001	p-value	< 0.001

Data are presented as median with interquartile range (IQR: 25th–75th percentiles). CMT difference and BCVA difference represent the absolute change from baseline to one month after the third intravitreal injection (three-month follow-up). Statistical significance was assessed using the Wilcoxon signed-rank test. CMT, Central Macular Thickness; BCVA, Best-Corrected Visual Acuity. P < 0.05 was considered statistically significant.

In parallel, a statistically significant functional improvement was also observed. Median BCVA improved from 0.30 (IQR: 0.15–0.50) at baseline to 0.40 (IQR: 0.25–0.50) at the three-month follow-up, with a median change of 0.00 (IQR: 0.00–0.20; P < 0.001), indicating that a meaningful proportion of eyes achieved measurable visual gains following the loading phase.

### 3.3 Factors influencing anti-VEGF treatment efficacy: GEE model analysis

To identify systemic and ocular predictors of anatomical response to Conbercept, both univariable and multivariable linear GEE models were constructed using the full dataset of 1,039 eyes. The results are presented in **Table 3**.

**Table 3:** Univariable and multivariable linear GEE models for factors associated with central macular thickness reduction following anti-VEGF treatment (N = 1,039 eyes).

Variable	Univariable GEE model			Multivariable GEE model		
	Estimate	Standard error	P-value	Estimate	Standard error	P-value
Gender	-0.197	8.371	0.981			
Age	-0.888	0.352	0.012	-0.199	0.195	0.307
Eye Laterality	-4.835	8.593	0.574			
Hypertension	-11.844	8.357	0.156			
CAD	0.277	10.324	0.978			
CMT baseline value	-0.728	0.027	<0.001	-0.778	0.050	<0.001
BCVA baseline value	454.36	21.965	<0.001	-70.220	27.631	0.011
TC	4.683	2.914	0.108			
TG	1.511	1.886	0.423			
HDL-C	7.667	12.395	0.536			
LDL-C	12.200	8.610	0.003	4.570	2.306	0.048
Glucose	3.697	0.781	<0.001	1.464	0.538	0.007
HbA1c	8.618	1.353	<0.001	4.174	1.001	<0.001

Estimates represent the regression coefficients from the GEE model with an exchangeable working correlation structure. Variables with P < 0.05 in the univariable model were entered into the multivariable model. Multicollinearity between fasting glucose and HbA1c was assessed prior to model construction, and the model was adjusted accordingly. A positive estimate indicates that higher values of the predictor are associated with a smaller reduction in CMT (i.e., poorer anatomical response). CAD, Coronary Artery Disease; TC, Total Cholesterol; TG, Triglycerides; HDL-C, High-Density Lipoprotein Cholesterol; LDL-C, Low-Density Lipoprotein Cholesterol; HbA1c, Glycated Hemoglobin A1c; CMT, Central Macular Thickness; BCVA, Best-Corrected Visual Acuity. P < 0.05 was considered statistically significant.

In the univariable GEE analysis, six variables demonstrated statistically significant associations with CMT change following treatment: age (Estimate = −0.888, P = 0.012), baseline CMT (Estimate = −0.728, P < 0.001), baseline BCVA (Estimate = 454.36, P < 0.001), LDL-C (Estimate = 12.200, P = 0.003), fasting blood glucose (Estimate = 3.697, P < 0.001), and HbA1c (Estimate = 8.618, P < 0.001). In contrast, gender, eye laterality, hypertension, CAD, TC, TG, and HDL-C did not reach statistical significance and were not carried forward into the multivariable model.

Following adjustment in the multivariable GEE model, five variables retained independent statistical significance as predictors of CMT reduction: baseline CMT (Estimate = −0.778, P < 0.001), baseline BCVA (Estimate = −70.220, P = 0.011), LDL-C (Estimate = 4.570, P = 0.048), fasting blood glucose (Estimate = 1.464, P = 0.007), and HbA1c (Estimate = 4.174, P < 0.001). Notably, age lost its independent significance after multivariable adjustment (P = 0.307), suggesting that its univariable association was likely confounded by other metabolic covariates. The positive estimates for HbA1c, fasting glucose, and LDL-C indicate that higher levels of these metabolic parameters were independently associated with a lesser degree of CMT reduction, implying poorer anatomical responsiveness to anti-VEGF therapy.

### 3.4 Comparison of baseline characteristics between responders and non-responders

An eye was classified as a “responder” if the percentage reduction in CMT was more than 10% from baseline at the three-month follow-up; all remaining eyes were designated as “non-responders.” Based on this criterion, 431 eyes (41.48%) were classified as responders and 608 eyes (58.52%) as non-responders. The comparative baseline characteristics and treatment outcomes of the two groups are presented in **Table 4**.

**Table 4:** Comparison of baseline characteristics and treatment outcomes between responders and non-responders.

Variable	Overall (N = 1039)	Responder (N = 431)	Non-responder (N = 608)	P-value
Age (years), median (p25 - p75)	60.000 (52.000 - 69.000)	62.000 (54.000 - 70.000)	59.000 (51.000 - 68.000)	0.007
Eye Laterality, n (p%)				0.922
Right eye	596.000 (57.363)	248.000 (57.541)	348.000 (57.237)	
Left eye	443.000 (42.637)	183.000 (42.459)	260.000 (42.763)	
Gender, n (p%)				0.998
Female	487.000 (46.872)	202.000 (46.868)	285.000 (46.875)	
male	552.000 (53.128)	229.000 (53.132)	323.000 (53.125)	
Hypertension, n (p%)	495.000 (47.642)	223.000 (51.740)	272.000 (44.737)	0.026
CAD, n (p%)	215.000 (20.693)	86.000 (19.954)	129.000 (21.217)	0.620
TC (mM), median (p25 - p75)	4.940 (4.110 - 5.890)	4.850 (4.030 - 5.960)	4.995 (4.200 - 5.860)	0.945
TG (mM), median (p25 - p75)	1.480 (1.030 - 2.290)	1.420 (1.000 - 2.350)	1.505 (1.050 - 2.250)	0.970
HDL-C (mM), median (p25 - p75)	1.160 (1.000 - 1.390)	1.170 (1.020 - 1.380)	1.145 (0.990 - 1.390)	0.682
LDL-C (mM), mean ± sd	3.190 ± 1.018	3.124 ± 1.077	3.238 ± 0.973	0.083
Glucose (mM), median (p25 - p75)	9.740 (6.880 - 14.135)	8.770 (6.420 - 13.070)	10.420 (7.240 - 14.665)	<0.001
HbA1c (%), mean ± sd	8.941 ± 2.997	8.082 ± 2.780	9.550 ± 2.999	<0.001
CMT baseline (μm), median (p25 - p75)	325.000 (281.000 - 448.000)	449.000 (361.000 - 573.000)	289.500 (268.000 - 319.000)	<0.001
CMT after treatment for three months (μm), median (p25 - p75)	291.000 (268.000 - 334.000)	288.500 (263.500 - 337.000)	292.000 (270.000 - 329.500)	0.072
CMT difference (μm), median (p25 - p75)	-17.000 (-100.000 - 4.000)	-130.000 (-246.000 - -73.000)	1.000 (-10.000 - 13.000)	<0.001
BCVA baseline, median (p25 - p75)	0.300 (0.150 - 0.500)	0.150 (0.100 - 0.250)	0.500 (0.300 - 0.500)	<0.001
BCVA after treatment for three months, median (p25 - p75)	0.400 (0.250 - 0.500)	0.400 (0.250 - 0.500)	0.400 (0.250 - 0.500)	0.169
BCVA difference, median (p25 - p75)	0.000 (0.000 - 0.200)	0.200 (0.100 - 0.350)	0.000 (0.000 - 0.000)	<0.001

CAD, Coronary Artery Disease; TC, Total Cholesterol; TG, Triglycerides; HDL-C, High-Density Lipoprotein Cholesterol; LDL-C, Low-Density Lipoprotein Cholesterol; HbA1c, Glycated Hemoglobin A1c; CMT, Central Macular Thickness; BCVA, Best-Corrected Visual Acuity. P < 0.05 was considered statistically significant.

The two groups were well-balanced in terms of gender distribution (female: 46.87% vs. 46.88%; P = 0.998) and eye laterality (right eye: 57.54% vs. 57.24%; P = 0.922), confirming the absence of systematic selection bias. Responders were significantly older than non-responders (median: 62.0 years, IQR: 54.0–70.0 vs. 59.0 years, IQR: 51.0–68.0; P = 0.007). A significantly higher proportion of responders had comorbid hypertension compared to non-responders (51.74% vs. 44.74%; P = 0.026), whereas the prevalence of CAD did not differ significantly between groups (19.95% vs. 21.22%; P = 0.620).

With respect to metabolic parameters, responders demonstrated significantly better glycemic control compared to non-responders, as reflected by a lower median fasting blood glucose (8.77 mM, IQR: 6.42–13.07 vs. 10.42 mM, IQR: 7.24–14.67; P < 0.001) and a lower mean HbA1c (8.08 ± 2.78% vs. 9.55 ± 3.00%; P < 0.001). No statistically significant between-group differences were observed for TC, TG, HDL-C, or LDL-C (all P > 0.05).

A critical and expected difference was observed in baseline CMT. Responders had a substantially higher baseline CMT than non-responders (median: 449.00 μm, IQR: 361.00–573.00 vs. 289.50 μm, IQR: 268.00–319.00; P < 0.001), reflecting the greater anatomical capacity for measurable improvement in eyes with more severe baseline edema. Conversely, responders had significantly lower baseline BCVA (median: 0.15, IQR: 0.10–0.25 vs. 0.50, IQR: 0.30–0.50; P < 0.001), consistent with the pattern that eyes with worse baseline visual function and greater structural compromise tend to exhibit more pronounced anatomical recovery. Following treatment, the median CMT reduction in responders was −130.00 μm (IQR: −246.00 to −73.00), compared to only +1.00 μm (IQR: −10.00 to 13.00) in non-responders (P < 0.001). Correspondingly, responders demonstrated a significantly greater improvement in BCVA (median change: 0.20, IQR: 0.10–0.35 vs. 0.00, IQR: 0.00–0.00; P < 0.001).

### 3.5 Identification of factors associated with poor anti-VEGF treatment response

To further delineate the independent determinants of poor anatomical response, a logistic GEE model was constructed with responder status (responder vs. non-responder) as the binary dependent variable. The results of the univariable and multivariable logistic GEE analyses are presented in **Table 5**.

**Table 5:** Univariable and multivariable logistic GEE analysis of factors associated with poor anti-VEGF treatment response .

Variable	Univariable logistic GEE model			Multivariable logistic GEE model		
	Estimate	Standard error	P-value	Estimate	Standard error	P-value
Gender	-0.001	0.126	0.998			
Age	-0.004	0.005	0.008	0.005	0.007	0.447
Eye Laterality	0.012	0.127	0.922			
Hypertension	-0.281	0.126	0.026	-0.279	0.173	0.106
CAD	0.077	0.156	0.620			
CMT baseline value	-0.014	0.001	<0.001	-0.008	0.002	<0.001
BCVA baseline value	9.621	0.552	<0.001	5.274	0.989	<0.001
TC	0.003	0.045	0.945			
TG	0.004	0.039	0.970			
HDL-C	-0.076	0.185	0.682			
LDL-C	0.111	0.065	0.087			
Glucose	0.057	0.014	<0.001	0.048	0.022	0.030
HbA1c	0.175	0.024	<0.001	0.192	0.035	<0.001

CAD, Coronary Artery Disease; TC, Total Cholesterol; TG, Triglycerides; HDL-C, High-Density Lipoprotein Cholesterol; LDL-C, Low-Density Lipoprotein Cholesterol; HbA1c, Glycated Hemoglobin A1c; CMT, Central Macular Thickness; BCVA, Best-Corrected Visual Acuity. P < 0.05 was considered statistically significant.

In the univariable logistic GEE analysis, age (P = 0.008), hypertension (P = 0.026), baseline CMT (P < 0.001), baseline BCVA (P < 0.001), fasting blood glucose (P < 0.001), and HbA1c (P < 0.001) were all significantly associated with responder status. Gender, eye laterality, CAD, TC, TG, HDL-C, and LDL-C did not achieve statistical significance.

In the multivariable logistic GEE model, four variables were identified as independent predictors of poor treatment response: baseline CMT (Estimate = −0.008, P < 0.001), baseline BCVA (Estimate = 5.274, P < 0.001), fasting blood glucose (Estimate = 0.048, P = 0.030), and HbA1c (Estimate = 0.192, P < 0.001). Age and hypertension lost statistical significance following multivariable adjustment (P = 0.447 and P = 0.106, respectively), suggesting that their univariable associations were largely attributable to confounding.

The direction and magnitude of these estimates warrant particular clinical attention. The negative estimate for baseline CMT indicates that eyes with lower baseline macular thickness were significantly more likely to be classified as non-responders, which is mechanistically consistent with the reduced capacity for measurable anatomical improvement in eyes with less severe edema. Conversely, the large positive estimate for baseline BCVA reflects that eyes with better baseline visual acuity—typically corresponding to less structurally compromised retinas—were substantially more likely to exhibit a poor anatomical response by the CMT-based criterion. Most critically, the positive and independently significant estimates for both HbA1c and fasting blood glucose confirm that poor systemic glycemic control is a robust independent risk factor for inadequate anatomical response to Conbercept, underscoring the integral role of metabolic status in modulating the local ocular response to anti-VEGF therapy.

## 4 Discussion

This large-scale retrospective cohort study, comprising 691 patients and 1,039 eyes, provides robust evidence identifying independent systemic and ocular predictors of anatomical response to intravitreal Conbercept in patients with DME. Our principal findings demonstrate that baseline CMT, baseline BCVA, LDL-C, fasting blood glucose, and HbA1c are independent predictors and significantly associated with of the degree of CMT reduction following the loading phase. Furthermore, the logistic GEE analysis revealed that lower baseline CMT, higher baseline BCVA, and elevated HbA1c and fasting glucose were the main factors independently associated with poor treatment response, defined as a failure to achieve a greater than 10% CMT reduction at three months. Collectively, these findings underscore a fundamental principle: the local anatomical response to anti-VEGF therapy in the eye is not an isolated ocular phenomenon, but is intrinsically modulated by the patient’s systemic metabolic milieu.

The most clinically compelling finding of this study is the robust and independent association between elevated HbA1c and poor anatomical response to Conbercept, which was consistently identified across both the linear GEE model for CMT reduction and the logistic GEE model for responder status. This finding is biologically coherent and mechanistically well-grounded. HbA1c serves as a reliable index of cumulative glycemic burden over the preceding two to three months, and chronic hyperglycemia perpetuates a pro-angiogenic retinal microenvironment through multiple converging pathways, including high glucose-induced oxidative stress, immune dysregulation, and vascular endothelial dysfunction (17). Sustained elevation of blood glucose drives the continuous overproduction of VEGF, thereby creating a state of persistent VEGF excess that may pharmacologically overwhelm or rapidly replenish the VEGF pool targeted by a three-injection loading course of anti-VEGF therapy; sustained VEGF-driven angiogenesis and inflammation represent the primary targets of current therapeutic strategies (18). Moreover, chronic hyperglycemia activates the protein kinase C (PKC) pathway, promotes advanced glycation end-product (AGE) accumulation, and induces oxidative stress, all of which independently compromise the integrity of the blood-retinal barrier and potentiate vascular permeability in ways that are not fully addressable by VEGF blockade alone (17, 18).

The clinical evidence surrounding this relationship, however, is not without nuance. A real-world analysis from the Fight Retinal Blindness! registry found no statistically significant association between baseline HbA1c and anti-VEGF outcomes at 24 months (19), potentially reflecting differences in follow-up duration, sample size, and the range of HbA1c values represented across study populations. The present study, with its larger cohort of 1,039 eyes and GEE-based adjustment for bilateral correlation, provides complementary evidence that elevated HbA1c independently predicts poor anatomical response in the Chinese clinical context. This interpretation is further corroborated by prospective observational data demonstrating that lower baseline HbA1c (≤7%) was independently associated with significantly greater reduction in central macular thickness at one month following anti-VEGF injection on both univariate and multivariate analyses (20). Furthermore, a multicenter retrospective study demonstrated that patients who actively engaged in glycemic management—through exercise and diet—following initiation of anti-VEGF therapy achieved significantly lower HbA1c at 6, 12, and 18 months compared with those who did not (21), suggesting that systemic metabolic optimization and ocular pharmacotherapy are synergistic rather than independent interventions. Taken together, our findings and the broader literature converge on a unified clinical message: elevated HbA1c is not merely a passive biomarker of disease severity, but an active modulator of the pharmacological efficacy of anti-VEGF therapy at the retinal level.

Beyond HbA1c, fasting blood glucose retained independent significance in the multivariable logistic GEE model after accounting for potential multicollinearity between the two glycemic indices. Although HbA1c and fasting glucose are inherently correlated, they capture distinct and complementary dimensions of glycemic dysregulation: HbA1c reflects chronic cumulative exposure, whereas fasting glucose captures the acute metabolic state at the time of treatment initiation (22). The independent significance of fasting glucose in the present analysis suggests that acute hyperglycemia at the perioperative period may exert an additional, non-redundant influence on the retinal vascular response to anti-VEGF injection, potentially through the acute upregulation of inflammatory cytokines and vasoactive mediators that transiently amplify vascular permeability (22). This observation has practical clinical implications. A treat-to-target framework for DME management, which proposes morphology-guided treatment algorithms to optimize and maintain vision outcomes (23), further underscores the importance of achieving favorable baseline metabolic and retinal conditions prior to initiating anti-VEGF loading therapy, suggesting that optimization of both chronic glycemic control and acute perioperative glucose levels may be warranted before commencing treatment.

Although anti-VEGF therapy remains the cornerstone of DME management (24), the interpretation of anatomical outcomes requires careful consideration of the response definition used. The finding that lower baseline CMT was independently associated with poor treatment response in the logistic GEE model should not be misconstrued as suggesting that less severely edematous eyes have inherently inferior pharmacological sensitivity to Conbercept. The anatomical floor effect is the primary explanation for this baseline CMT-related finding and largely accounts for the observed association. This well-recognized methodological phenomenon in DME research dictates that eyes with substantially elevated baseline CMT possess considerably greater arithmetic capacity for measurable thickness reduction, making it far more likely that they will surpass the 10% CMT reduction threshold required for responder classification. Conversely, eyes approaching baseline CMT values near the lower boundary of the study inclusion criterion (250 μm) have a markedly constrained range of possible downward change, rendering the achievement of a 10% reduction structurally difficult regardless of the pharmacological efficacy of the administered agent. This interpretation is supported by the marked difference in median baseline CMT between responders (449.0 μm) and non-responders (289.5 μm) observed in **Table 4**, which strongly suggests that the apparent association between low baseline CMT and non-response is largely driven by the mathematical constraints of the response definition rather than genuine treatment refractoriness. This finding highlights an important limitation of overly simplistic binary CMT-based response classifications. A secondary analysis of three landmark DRCR Retina Network RCTs proposed contextualized, baseline-adjusted thresholds to define strong versus weak anatomical responses, underscoring that uniform binary criteria fail to capture the inherent heterogeneity of DME cohorts and advocating for response phenotyping that accounts for baseline structural severity (25). These considerations support the increasing adoption of proportional or continuous outcome measures in future DME efficacy research.

The observation that higher baseline BCVA was strongly and independently associated with classification as a non-responder in the logistic GEE model reflects a well-established structural-functional dissociation pattern in DME. Eyes with better preserved baseline visual acuity typically harbor less severe retinal structural compromise, including less extensive disorganization of the retinal inner layers (DRIL), better preservation of the ellipsoid zone, and less severe outer nuclear layer thinning (13). Such eyes, while possessing relatively good functional reserve, often present with more modest degrees of macular thickening, and therefore face the same anatomical floor effect described above when assessed by CMT-based response criteria. Simultaneously, eyes with poor baseline BCVA tend to have higher baseline CMT, greater structural disorganization, and consequently, more pronounced anatomical improvement following anti-VEGF loading—often meeting the 10% CMT reduction threshold with considerably greater ease. The strong positive coefficient for baseline BCVA in the logistic GEE model (Estimate = 5.274) is therefore a reflection of this interdependency between structural and functional baseline status, rather than an indication that better visual function at baseline portends worse therapeutic outcomes per se. These findings are consistent with prior observations from Protocol T and related DRCR.net analyses, in which baseline visual acuity was a significant effect modifier of anti-VEGF treatment response (9).

The identification of LDL-C as an independent predictor of CMT reduction in the linear GEE model—though not achieving independent significance in the multivariable logistic GEE model—suggests that elevated LDL-C may modulate the quantitative degree of macular edema resolution without exerting a sufficiently strong effect to alter the binary probability of achieving the 10% CMT reduction threshold. This gradient-level rather than threshold-level effect is biologically plausible. Dyslipidemia, particularly elevated LDL-C, is independently and nonlinearly associated with increased DME probability, with a positive association identified up to an inflection point of approximately 4.85 mmol/L (OR = 1.60, 95% CI: 1.10–2.34) (26). Mechanistically, LDL promotes retinal endothelial dysfunction through its conversion to oxidized LDL (OxLDL), which activates inflammatory cascades including the NF-κB pathway and disrupts tight junction proteins such as ZO-1, collectively increasing retinal microvascular permeability in ways that may attenuate the full anatomical resolution achievable with anti-VEGF monotherapy alone. Lipid deposition in the form of hard exudates is a recognized morphological manifestation of severe dyslipidemia in DME, and these deposits represent a structural impediment to photoreceptor recovery that anti-VEGF therapy alone cannot resolve. The present data, when considered alongside evidence from the LENS trial demonstrating that fenofibrate reduced DR progression risk by 27% (HR = 0.73, 95% CI: 0.58–0.91) and macular edema incidence by 50% (HR = 0.50, 95% CI: 0.30–0.84) compared with placebo (27), and supported by mechanistic evidence from the FIELD and ACCORD-EYE studies reviewed through the LENS trial framework (28), lend further support to the concept that comprehensive lipid management should be integrated into the multimodal treatment strategy for DME patients with elevated LDL-C.

Notably, hypertension and coronary artery disease (CAD), despite being prevalent comorbidities in the present cohort (48.19% and 19.97%, respectively), did not emerge as independent predictors of anti-VEGF treatment response in the multivariable models. While hypertension is well-established as a risk factor for DME development and progression (5), its independent contribution to short-term anatomical anti-VEGF outcomes—after adjustment for glycemic and lipid parameters—has been inconsistent across studies, with some real-world analyses suggesting that metabolic covariates such as HbA1c and lipid profiles may account for the majority of the variance in treatment response (20, 21). One possible explanation is that hypertension may exert its pathological influence primarily through chronically mediated structural damage to retinal microvasculature that precedes treatment, rather than through dynamic modulation of the acute pharmacological response to anti-VEGF loading. Additionally, the confounding between hypertension and other metabolic comorbidities in a T2DM population may have attenuated its independent signal following multivariable adjustment. These null findings should be interpreted with caution and do not preclude a clinically meaningful role for blood pressure control in long-term DME management (5).

A key methodological strength of this study is the application of GEE models to account for the inherent intra-subject correlation between bilateral eyes. The failure to account for this dependency in standard regression analyses systematically underestimates standard errors and inflates Type I error rates, a statistical pitfall that has been documented extensively in the ophthalmic epidemiology literature (15). By employing an exchangeable working correlation structure within the GEE framework, the present analysis yields statistically valid and robust estimates of predictor effects while maximizing the analytical utilization of the full bilateral dataset. Additionally, the large cohort size of 1,039 eyes provides substantial statistical power for the detection of moderate effect sizes, lending confidence to the stability of the multivariable model estimates. The use of a standardized loading-phase treatment protocol—three consecutive monthly injections of Conbercept—across all patients further minimizes treatment heterogeneity as a source of confounding in the outcome assessment.

Several limitations of the present study merit acknowledgment. First, the retrospective cohort design precludes causal inference, and unmeasured confounding from variables not captured in the electronic medical record—most notably diabetes duration and baseline diabetic retinopathy severity grade—cannot be excluded. Diabetes duration is a well-established determinant of DME severity and treatment response, and its absence from the analytical model represents a meaningful limitation that warrants attention in future prospective studies (5). Diabetes duration is a well-established determinant of DME severity and treatment response; prolonged diabetes contributes to irreversible retinal microvascular lesions and impairs the efficacy of anti-VEGF therapy. Standardized baseline diabetic retinopathy grading was also unavailable for all subjects. The absence of these two key variables from our analytical model represents a significant limitation, and we recommend that future prospective studies fully collect and adjust for diabetes duration and DR severity grade to optimize model robustness. Second, the three-month follow-up period was designed to evaluate the anatomical response to the standardized loading phase of Conbercept. However, this is a short-term observation window. It cannot assess the long-term durability of treatment effects, nor capture the treatment outcomes of late responders who achieve gradual edema resolution during subsequent maintenance therapy. In addition, this time frame is inadequate to analyze the need for rescue therapy and sustained anatomical remission over 12 or 24 months. Longer follow-up is required to characterize late treatment response and long-term therapeutic effects. Third, we adopted a binary definition of treatment response based on a 10% reduction in CMT, which introduces inevitable response definition bias. the binary CMT-based responder definition, while pragmatic, is subject to the anatomical floor effect described above and may misclassify eyes with clinically meaningful but sub-threshold responses as non-responders, a limitation that contextualized baseline-adjusted response thresholds have been proposed to address (25). Fourth, the study was conducted at a single Chinese tertiary center using Conbercept exclusively, which may limit the generalizability of findings to settings employing other anti-VEGF agents or to more ethnically diverse populations. Fifth, several key ocular confounding factors associated with anti-VEGF treatment response were not collected and adjusted for in this study, including integrity of the inner segment–outer segment junction, presence of mild epiretinal membranes, status of retinal inner layer disorganization, prior ocular inflammation and prior retinal treatment history. The lack of these variables may introduce residual confounding and influence the stability of correlation results.

The findings of this study carry direct and actionable clinical implications for the management of DME in real-world practice, but they should be interpreted as hypothesis-generating. The observed associations of HbA1c and fasting glucose with poor short-term anatomical response support multidisciplinary collaboration between ophthalmologists and endocrinologists before and during anti-VEGF therapy. Specifically, our data suggest that patients presenting with HbA1c levels substantially above therapeutic targets may represent a high-risk subgroup in whom intensification of systemic glycemic management prior to or concurrent with anti-VEGF loading could meaningfully enhance anatomical outcomes. Similarly, the gradient-level association between LDL-C and CMT reduction supports the routine assessment and active management of lipid profiles as an adjunctive strategy in DME patients, particularly in light of evidence from the LENS trial demonstrating that fenofibrate reduces DR progression risk and macular edema incidence by clinically meaningful magnitudes (27, 28). Prospective studies incorporating pre-treatment metabolic optimization interventions, longer follow-up, and validated baseline-adjusted response criteria are warranted to determine whether systemic metabolic control can improve the real-world efficacy of anti-VEGF therapy in DME.

## 5 Conclusion

In summary, this large-scale retrospective cohort study of 691 patients and 1,039 eyes demonstrates that systemic metabolic control and baseline ocular morphology are key factors independently associated with anatomical response to intravitreal Conbercept in patients with DME. Our findings establish that elevated HbA1c and LDL-C levels, along with a lower baseline CMT, are independent predictors and significantly correlated with poor anatomical improvement following a standard three-month loading phase of anti-VEGF therapy. In the logistic GEE analysis, elevated HbA1c and fasting blood glucose emerged as the most robust systemic risk factors associated with non-response, underscoring the critical role of the systemic metabolic milieu in modulating the local pharmacological efficacy of intravitreal anti-VEGF agents. Future prospective studies are needed to evaluate whether targeted pre-treatment metabolic optimization—encompassing both glycemic and lipid control—can translate into measurable improvements in anatomical and functional outcomes in DME patients undergoing anti-VEGF loading therapy.

## Data Availability

The original contributions presented in the study are included in the article/supplementary material. Further inquiries can be directed to the corresponding author.
